# A Nonclassical Mechanism of β-Lactam Resistance in Methicillin-Resistant Staphylococcus aureus and Its Effect on Virulence

**DOI:** 10.1128/spectrum.02284-22

**Published:** 2022-10-31

**Authors:** Nidhi Satishkumar, Li-Yin Lai, Nagaraja Mukkayyan, Bruce E. Vogel, Som S. Chatterjee

**Affiliations:** a Department of Microbial Pathogenesis, School of Dentistry, University of Maryland, Baltimore, Maryland, USA; b Institute of Marine and Environmental Technology (IMET), Baltimore, Maryland, USA; c Center for Biomedical Engineering and Technology, University of Maryland School of Medicine, University of Maryland, Baltimore, Maryland, USA; Health Canada

**Keywords:** PBP4, *Staphylococcus aureus*, beta-lactam resistance, virulence

## Abstract

Methicillin-resistant Staphylococcus aureus (MRSA) is a group of pathogenic bacteria that are infamously resistant to β-lactam antibiotics, a property attributed to the *mecA* gene. Recent studies have reported that mutations associated with the promoter region of *pbp4* demonstrated high levels of β-lactam resistance, suggesting the role of PBP4 as an important non-*mecA* mediator of β-lactam resistance. The *pbp4*-promoter-associated mutations have been detected in strains with or without *mecA*. Our previous studies that were carried out in strains devoid of *mecA* described that *pbp4*-promoter-associated mutations lead to PBP4 overexpression and β-lactam resistance. In this study, by introducing various *pbp4*-promoter-associated mutations in the genome of a MRSA strain, we demonstrate that PBP4 overexpression can supplement *mecA*-associated resistance in S. aureus and can lead to increased β-lactam resistance. The promoter and regulatory region of *pbp4* is shared with a divergently transcribed gene, *abcA*, which encodes a multidrug exporter. We demonstrate that the promoter mutations caused an upregulation of *pbp4* and downregulation of *abcA*, confirming that the resistant phenotype is associated with PBP4 overexpression. PBP4 has also been associated with staphylococcal pathogenesis, however, its exact role remains unclear. Using a Caenorhabditis elegans model, we demonstrate that strains having increased PBP4 expression are less virulent than wild-type strains, suggesting that β-lactam resistance mediated via PBP4 likely comes at the cost of virulence.

**IMPORTANCE** Our study demonstrates the ability of PBP4 to be an important mediator of β-lactam resistance in not only methicillin-susceptible Staphylococcus aureus (MSSA) background strains as previously demonstrated but also in MRSA strains. When present together, PBP2a and PBP4 overexpression can produce increased levels of β-lactam resistance, causing complications in treatment. Thus, this study suggests the importance of monitoring PBP4-associated resistance in clinical settings, as well as understanding the mechanistic basis of associated resistance, so that treatments targeting PBP4 may be developed. This study also demonstrates that S. aureus strains with increased PBP4 expression are less pathogenic, providing important hints about the role of PBP4 in S. aureus resistance and pathogenesis.

## INTRODUCTION

Staphylococcus aureus is a Gram-positive pathogen that can cause skin and soft tissue infections (SSTIs), bacteremia, osteomyelitis, and sepsis in humans (1). Along with being equipped with a wide array of virulence factors, S. aureus is also resistant to a wide range of antibiotics, making infections difficult to treat (2). In particular, methicillin-resistant Staphylococcus aureus (MRSA) is infamous for being resistant to β-lactams, resulting in over 120,000 deaths globally in 2019 (3).

β-Lactams are a class of antibiotics known for their safety, efficacy, and tissue distribution which makes them the most commonly prescribed antibiotics (4). β-Lactams bind to penicillin binding proteins (PBPs), which are integral proteins involved in the final stages of cell wall synthesis. The binding of β-lactams to PBPs causes their inactivation, leading to weakening of the cell wall and, subsequently, cell death (5). MRSA contains the gene *mecA*, which encodes PBP2a, a PBP that has decreased affinity toward β-lactams, allowing cells to survive even in high concentrations of β-lactams (2).

Historically, resistance mechanisms in S. aureus have been acquired in waves. With the introduction of every new generation of β-lactams, S. aureus has been able to develop new resistance mechanisms (2). This ability of S. aureus to constantly develop resistance mechanisms makes it important to focus on other, novel mechanisms of resistance. Keeping this in mind, our previous studies involved serial passaging of strains in increasing amounts of β-lactams with the aim of identifying non-*mecA* mechanisms of resistance (6). We determined that mutations associated with the promoter region of PBP4 were largely prevalent in resistant strains (6, 7). Various studies by other groups also identified *pbp4*-associated mutations in laboratory-generated (8, 9) as well as in clinically isolated (10, 11) resistant strains of both MRSA and MSSA backgrounds, suggesting the clinical relevance of these mutations. PBP4, a nonessential, low-molecular-weight (LMW) PBP in S. aureus, is produced in small amounts, and its role in resistance or pathogenesis is not very well described (12). We previously demonstrated that *pbp4*-promoter-associated mutations led to significant β-lactam resistance in strains devoid of *mecA* by causing increased PBP4 expression and enhanced cell wall cross-linking (7). However, the effect of these mutations in strains containing *mecA* is unknown. Thus, in this study, we inspected whether PBP4, through the means of overexpression, supplemented *mecA*-mediated β-lactam resistance. We used the strain SF8300, a derivative of USA300, which is one of the most prominent community-associated MRSA (CA-MRSA) strains detected in the United States (2). *pbp4*-promoter-associated mutations that were detected in a laboratory-passaged resistant strain that was devoid of *mecA* (namely, CRB) and in a resistant strain containing *mecA* (namely, CRT) were introduced in SF8300 (6). Furthermore, a *pbp4*-promoter-associated mutation detected in a β-lactam-resistant clinical isolate (namely, Strain 1) was also used in this study (13). Using these promoter mutants, we demonstrated that *pbp4*-promoter-associated mutations lead to PBP4 overexpression and increased β-lactam resistance in MRSA strains in a mechanism similar to what was previously shown in MSSA strains (7, 13).

*pbp4* shares its promoter and regulatory region with *abcA*, a gene that encodes an ATP-binding cassette transporter protein. ABC transporters are notorious for enabling resistance to chemotherapeutic agents in both prokaryotes and eukaryotes via export activity. Since AbcA has previously been associated with antibiotic resistance (14), we were interested in understanding whether the promoter-associated mutations altered *abcA* expression that potentially led to AbcA-mediated β-lactam resistance. However, our results indicated that while the promoter-associated mutations led to increased *pbp4* expression, they resulted in downregulation of *abcA*, indicating that AbcA did not have any role in β-lactam resistance and that the resistance phenotype observed in the promoter mutants was attributed to PBP4.

Along with β-lactam resistance, PBP4 has recently been associated with virulence in previous studies. However, the exact role of PBP4 in virulence remains unclear, as these studies had contrasting reports (15, 16). In order to get a better understanding of the role of PBP4 in virulence, we used a Caenorhabditis elegans infection model and demonstrated that MRSA strains containing *pbp4*-promoter-associated mutations had decreased virulence compared with the wild-type (WT) strain. We also generated *abcA* mutant strains to demonstrate that *abcA* downregulation due to the promoter mutations did not result in the observed resistance and virulence phenotypes.

Our findings suggest the importance of monitoring PBP4-associated resistance in both MRSA and MSSA strains and indicate that treatment options would potentially have to consider targeting both PBP2a and PBP4, as when present, both the proteins independently mediate β-lactam resistance via distinct mechanisms leading to increased resistance. Our results also confirm that promoter-associated mutations only allow for PBP4 overexpression and do not facilitate AbcA-mediated resistance, indicating that PBP4-associated resistance has the potential of being a prominent resistance mechanism in the future. Finally, our results also provide important clues associated with the role of PBP4 in virulence and suggest that strains with increased PBP4 may have decreased virulence, a phenomenon that needs to be studied further in detail in the future.

## RESULTS

### *pbp4*-promoter-associated mutations led to increased PBP4 expression and β-lactam resistance in MRSA strains.

**FIG 1:**
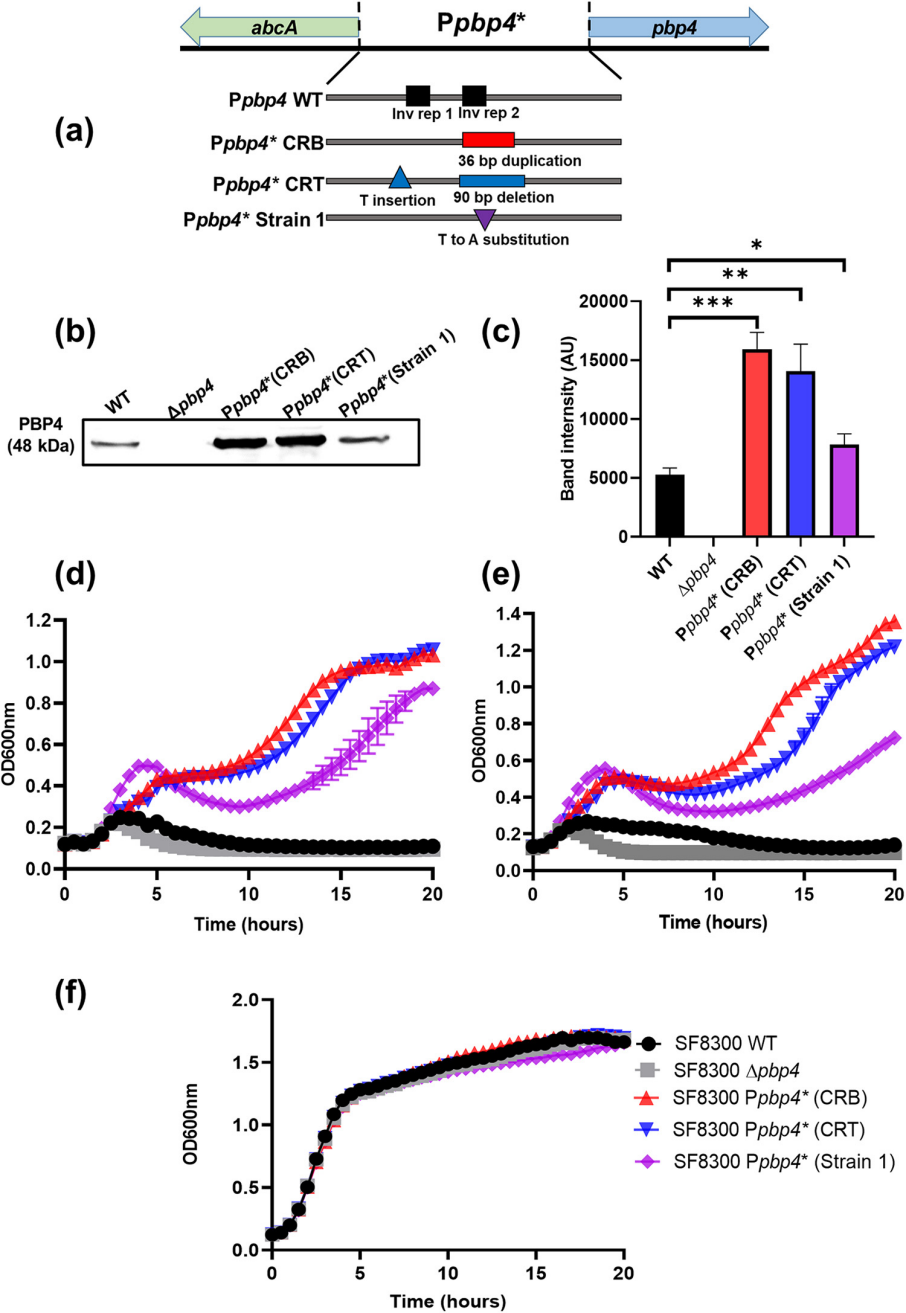
*pbp4*-promoter-associated mutations caused PBP4 overexpression and increased β-lactam resistance in MRSA strains. (a) Schematic diagram of the *pbp4*-P*pbp4*-*abcA* region. The *pbp4* and *abcA* transcriptional start sites are separated by 420 bp of the promoter region. Along with the wild-type promoter (P*pbp4* WT), promoter mutations seen in the strain CRB (36-bp duplication at 290 bp upstream the start codon), CRT (T insertion at 377 bp and a 90-bp deletion at 275 bp upstream the start codon), and Strain 1 (T-to-A substitution 266 bp upstream the start codon) are represented. (b) Immunoblotting of PBP4 expression levels among selected strains. Proteins from the membrane fraction of the WT strain (SF8300) and strains containing promoter-associated mutations (SF8300 Ppbp4* [CRB], SF8300 Ppbp4* [CRT], and SF8300 Ppbp4* [Strain 1]) were probed with an antibody specific to PBP4 and were visualized for protein expression. (c) Densitometry analysis of PBP4 immunoblotting. Compared with the WT, the strains with promoter mutations had increased levels of PBP4 (48 kDa). Δ*pbp4* was used as a control, where there was no PBP4 band detected. (SF8300 WT versus SF8300 Ppbp4* [CRB], *P* = 0.0003; SF8300 WT versus SF8300 Ppbp4* [CRT], *P* = 0.0030 and SF8300 WT versus SF8300 Ppbp4* [Strain 1], *P* = 0.0141). Band intensity is represented as arbitrary units (AU). (d) Growth assay of SF8300 WT and mutant strains in the presence of 4 μg/mL nafcillin. Strains containing promoter-associated mutations showed significantly enhanced survival compared with the WT and Δ*pbp4* strains. (SF8300 WT versus SF8300 Ppbp4* [CRB], SF8300 WT versus SF8300 Ppbp4* [CRT], and SF8300 WT versus SF8300 Ppbp4* [Strain 1], *P* < 0.0001). (e) Growth assay of SF8300 WT and mutant strains in the presence of 8 μg/mL oxacillin. Strains containing promoter-associated mutations showed significantly enhanced survival compared with the WT and Δ*pbp4* strains. (SF8300 WT versus SF8300 Ppbp4* [CRB], SF8300 WT versus SF8300 Ppbp4* [CRT], and SF8300 WT versus SF8300 Ppbp4* [Strain 1], *P* < 0.0001). (f) Growth assay of SF8300 WT and mutant strains in the absence of antibiotics. There was no significant difference in growth pattern among the selected isogenic strains in the absence of antibiotics.

Our previous studies demonstrated that *pbp4*-promoter-associated mutations led to PBP4 overexpression that subsequently resulted in β-lactam resistance (7, 13). These studies were performed by introducing promoter-associated mutations into strains devoid of *mecA*. Using allelic replacement, we created isogenic mutants by introducing three different, previously detected mutations in the *pbp4* promoter region of SF8300 (13, 17). Of these mutants, two mutations were detected in laboratory-generated resistant strains as a result of a passaging experiment. The first mutation was detected in the strain CRB and was a 36-bp duplication 290 bp upstream of the *pbp4* start codon. This mutation was introduced into the *pbp4*-promoter region of SF8300, giving rise to the strain SF8300 Ppbp4* (CRB). Similarly, insertion of mutations detected in the strain CRT (a thymine insertion 377 bp upstream the start codon and a 90-bp deletion 275 bp upstream the start codon) into the *pbp4*-promoter region of SF8300 produced SF8300 Ppbp4* (CRT). The third mutation was one detected in a clinically isolated strain (10, 13), namely, Strain 1, and consisted of a T-to-A substitution 266 bp upstream of the start codon. The introduction of this mutation in SF8300 gave rise to SF8300 Ppbp4* (Strain 1) (Fig. 1a).

In order to determine if the introduction of these mutations affected PBP4 expression in the selected isogenic strains, immunoblotting was performed using an antibody specific to PBP4. Compared with the wild-type (WT) strain SF8300, strains containing promoter-associated mutations had a significantly increased expression of PBP4 (Fig. 1b; see Fig. S1 in the supplemental material). SF8300 Ppbp4* (CRB) had the largest amount of expressed protein, followed by SF8300 Ppbp4* (CRT) and SF8300 Ppbp4* (Strain 1). Δ*pbp4* was used as a control, for which there was no PBP4 band detected (Fig. 1c). These results indicated that PBP4 overexpression occurred as a result of promoter-associated mutations in MRSA strains, in a manner similar to what was previously detected in MSSA strains (7, 13).

Following confirmation of PBP4 overexpression, we performed a growth assay in the presence of β-lactams, such as nafcillin and oxacillin, to examine the effect of PBP4 overexpression on β-lactam resistance. When exposed to β-lactams, strains with promoter-associated mutations were able to survive significantly better in the presence of either of the antibiotics than the WT and Δ*pbp4* strains for 4 μg/mL nafcillin (Fig. 1d) and 8 μg/mL oxacillin (Fig. 1e). The wild-type strain as well as the strains with promoter-associated mutations all had similar growth patterns in the absence of β-lactams (Fig. 1f). Taken together, these results suggested that promoter-associated mutations led to PBP4 overexpression and β-lactam resistance in the MRSA background, similar to what was previously demonstrated in MSSA background strains (7, 13).

### *pbp4*-promoter-associated mutations led to increased expression of *pbp4* and decreased expression of *abcA*.

**FIG 2:**
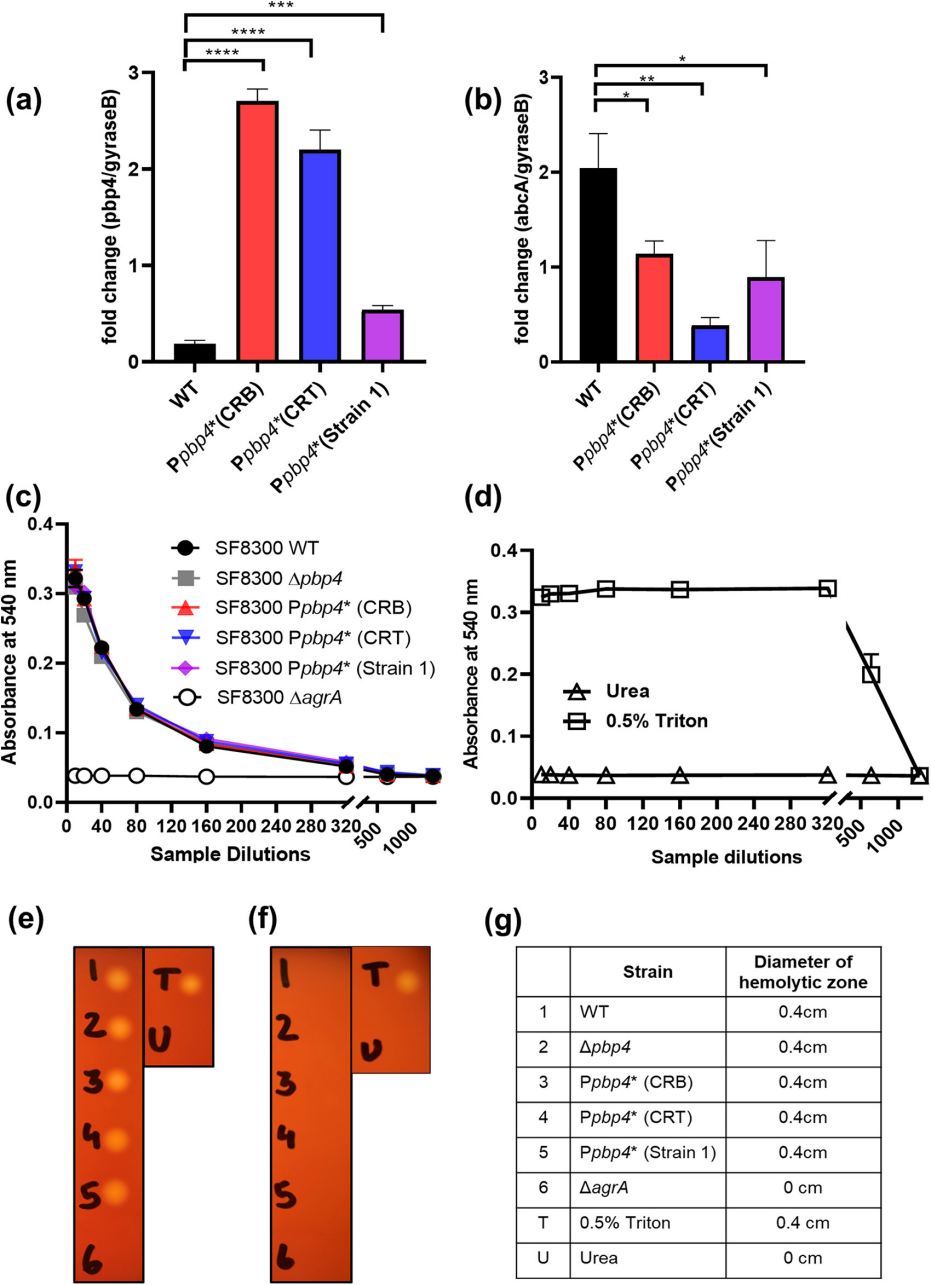
Promoter mutations cause an upregulation of *pbp4* and downregulation of *abcA.* (a) qRT-PCR analysis of *pbp4*. *pbp4* transcriptional expression was significantly increased in strains containing promoter-associated mutations compared with the WT strain (SF8300 WT versus SF8300 Ppbp4* [CRB], *P* < 0.0001; SF8300 WT versus SF8300 Ppbp4* [CRT], *P* < 0.0001; SF8300 WT versus SF8300 Ppbp4* [Strain 1], *P* = 0.0004). (b) qRT-PCR analysis of *abcA*. *abcA* transcriptional expression was significantly decreased in strains containing promoter-associated mutations compared with the WT strain. (SF8300 WT versus SF8300 Ppbp4* [CRB], *P* = 0.0161; SF8300 WT versus SF8300 Ppbp4* [CRT], *P* = 0.0016; SF8300 WT versus SF8300 Ppbp4* [Strain 1], *P* = 0.0203). (c) Hemolysis assay. Analysis of the ability of butanol-extracted phenol-soluble modulins (PSMs) to lyse sheep erythrocytes was carried out using a hemolysis assay. There was no difference detected in the hemolytic abilities among the WT strain and strains containing promoter-associated mutations. Δ*agrA* was used as a control, which showed no hemolytic activity. (d) Controls used for a hemolysis assay of sheep erythrocytes. A hemolysis assay of decreasing concentrations of 8 M urea indicated that urea in itself did not have any hemolytic ability. A total of 0.5% Triton X-100 was used as a positive control, which showed maximum hemolysis. An analysis of the hemolytic ability of butanol-extracted phenol-soluble modulins (PSMs) by spotting them onto blood agar-TSA plates. There was no difference in hemolysis among WT and promoter mutant strains when PSMs were diluted 10 times (e) or diluted 100 times (f). See Figure 2g for strain legend. (g) Diameter of the hemolytic zone detected on the blood agar-TSA plates for PSMs from the selected strains.

The *pbp4* gene shares its 420-bp promoter and regulatory region with a neighboring, divergently transcribed gene, namely, *abcA* (18). AbcA is an ATP binding cassette-like transporter protein that has been reported to export various chemicals, dyes, and antibiotics, thus contributing to antibiotic resistance in S. aureus (14, 19). It also has the ability to export phenol-soluble modulins (PSMs) that are cytolytic toxins and, thus, also plays a role in S. aureus virulence (20). Since the mutations detected upstream of the *pbp4* start codon also lie upstream of the *abcA* start codon (Fig. 1a), we were interested in whether they caused any alterations in the expression of *abcA* that subsequently contributed to β-lactam resistance in S. aureus. We thus performed reverse transcription-quantitative PCR (qRT-PCR) to examine the expression pattern of *pbp4* and *abcA* in the presence of promoter-associated mutations. At 4 h, *pbp4* transcripts were expressed in very small amounts in the SF8300 WT and were significantly increased in strains containing promoter-associated mutations (Fig. 2a) (SF8300 P*pbp4* WT versus SF8300 Ppbp4* [CRB], *P* < 0.0001; SF8300 WT versus SF8300 Ppbp4* [CRT], *P* < 0.0001; SF8300 WT versus SF8300 Ppbp4* [Strain 1], *P* = 0.0004). On the other hand, *abcA* transcripts had increased expression in SF8300 WT but had significantly decreased expression in strains containing promoter-associated mutations (Fig. 2b) (SF8300 WT versus SF8300 Ppbp4* [CRB], *P* = 0.0161; SF8300 WT versus SF8300 Ppbp4* [CRT], *P* = 0.0016; SF8300 WT versus SF8300 Ppbp4* [Strain 1], *P* = 0.0203). These results indicate that the promoter-associated mutations caused overexpression of *pbp4* but led to a downregulation of *abcA*. Thus, the increased β-lactam resistance seen due to the promoter-associated mutations was likely to be solely attributed to PBP4 overexpression and not AbcA activity. The results of qRT-PCR also corresponded with the results seen with PBP4 immunoblotting in terms of different levels of PBP4 overexpression, which indicated that SF8300 Ppbp4* (CRB) had the largest amount of PBP4 overexpression, followed by SF8300 Ppbp4* (CRT) and SF8300 Ppbp4* (Strain 1) (Fig. 1c, Fig. 2a).

In order to determine whether the promoter mutations affected the expression of genes downstream of *abcA* or *pbp4*, we also performed qRT-PCR analysis of genes flanking *abcA* (*nupC2*) and *pbp4* (*tagD*) (see Fig. S2a in the supplemental material). The results indicated that there was no significant difference between the expression of *nupC2* (Fig. S2b) or *tagD* (Fig. S2c) among the WT strain and the promoter mutants, suggesting that the promoter mutants caused changes only in expression of *pbp4* and *abcA* (SF8300 WT versus SF8300 Ppbp4* [CRB], SF8300 WT versus SF8300 Ppbp4* [CRT], SF8300 WT versus SF8300 Ppbp4* (Strain 1), *P* = not significant [ns]).

One of the important functions performed by AbcA is the export of cytolytic toxins, such as phenol-soluble modulins (PSMs) that can lyse erythrocytes and neutrophils (20). In order to determine the effect of AbcA downregulation and its subsequent effect on its activity, we measured the amount of PSMs exported by WT cells and cells that contained the promoter-associated mutations. Using 1-butanol, PSMs were extracted from culture supernatants and were resuspended in urea, following which their ability to lyse sheep erythrocytes was measured by performing hemolysis assays as previously shown (21). It was seen that none of the strains containing promoter-associated mutations differed in their hemolytic capabilities compared with the WT strain, as they all showed equal amounts of hemolysis at the different dilutions used (Fig. 2c). A Δ*agrA* strain was used as a negative control, which showed no hemolysis at all. Urea was used as a control, which by itself did not have any hemolytic abilities, whereas 0.5% Triton X-100 was used as a positive control that showed maximum hemolysis (Fig. 2d). Along with using sheep blood for the hemolysis assay, 10× and 100× dilutions of the extracted PSMs were spotted onto blood-tryptic soy agar (TSA) plates to analyze differences in hemolysis levels (Fig. 2e to g). The pattern of hemolysis seen on the plates also indicated that there was no difference in the hemolytic abilities between the WT and the mutant strains. The Δ*agrA*, urea, and Triton X-100 controls were used here, too. Together, these results confirmed that the β-lactam-resistant phenotypes seen with promoter-associated mutations were due to PBP4 overexpression and that AbcA had no role in it.

To further confirm that *abcA* downregulation did not cause the resistance phenotypes (Fig. 1d and e), we generated *abcA* mutants in the *mecA*- and *blaZ*-devoid derivatives of SF8300 called SF8300ex. SF8300 contains the *blaZ* plasmid, which also contains *ermB*, an erythromycin resistant gene (22). In order to accommodate for the selection of transposon insertion mutants based on erythromycin resistance, we had to use a strain devoid of the *blaZ* plasmid. Eviction of this plasmid results in the constitutive expression of *mecA*, as the regulatory system associated with *blaZ* expression (namely, the BlaR1-BlaI system) also regulates *mecA* in USA300 (23). In order to avoid the effect of constitutive expression of *mecA*, we used SF8300ex, a strain devoid of both *blaZ* and *mecA.* An *abcA* insertional transposon was introduced in the wild-type strain (SF8300ex or WTex) to generate SF8300ex *abcA*::Tn and in a representative promoter mutant to generate SF8300ex Ppbp4* (CRB) *abcA*::Tn. Phenotypic verification of these strains was performed by qRT-PCR analysis for *pbp4* and *abcA* (see Fig. S3a and S3b in the supplemental material) and Bocillin assay (Fig. S3c and S3d).

Growth assays for these strains with 0.25 μg/mL nafcillin or 0.5 μg/mL oxacillin indicated that the *abcA* mutant did not affect the phenotypes of the strains, as both the strains containing promoter mutants grew significantly better than strains with wild-type promoters (see Fig. S4 in the supplemental material). For 0.25 μg/mL nafcillin, the SF8300ex *abcA*::Tn and SF8300ex Ppbp4*(CRB) *abcA*::Tn *P* value was <0.0001. For 0.5 μg/mL oxacillin, the SF8300ex *abcA*::Tn and SF8300ex Ppbp4*(CRB) *abcA*::Tn *P* value was 0.0075.

### S. aureus strains with *pbp4*-promoter-associated mutations were less virulent to C. elegans than the wild-type strain.

**FIG 3:**
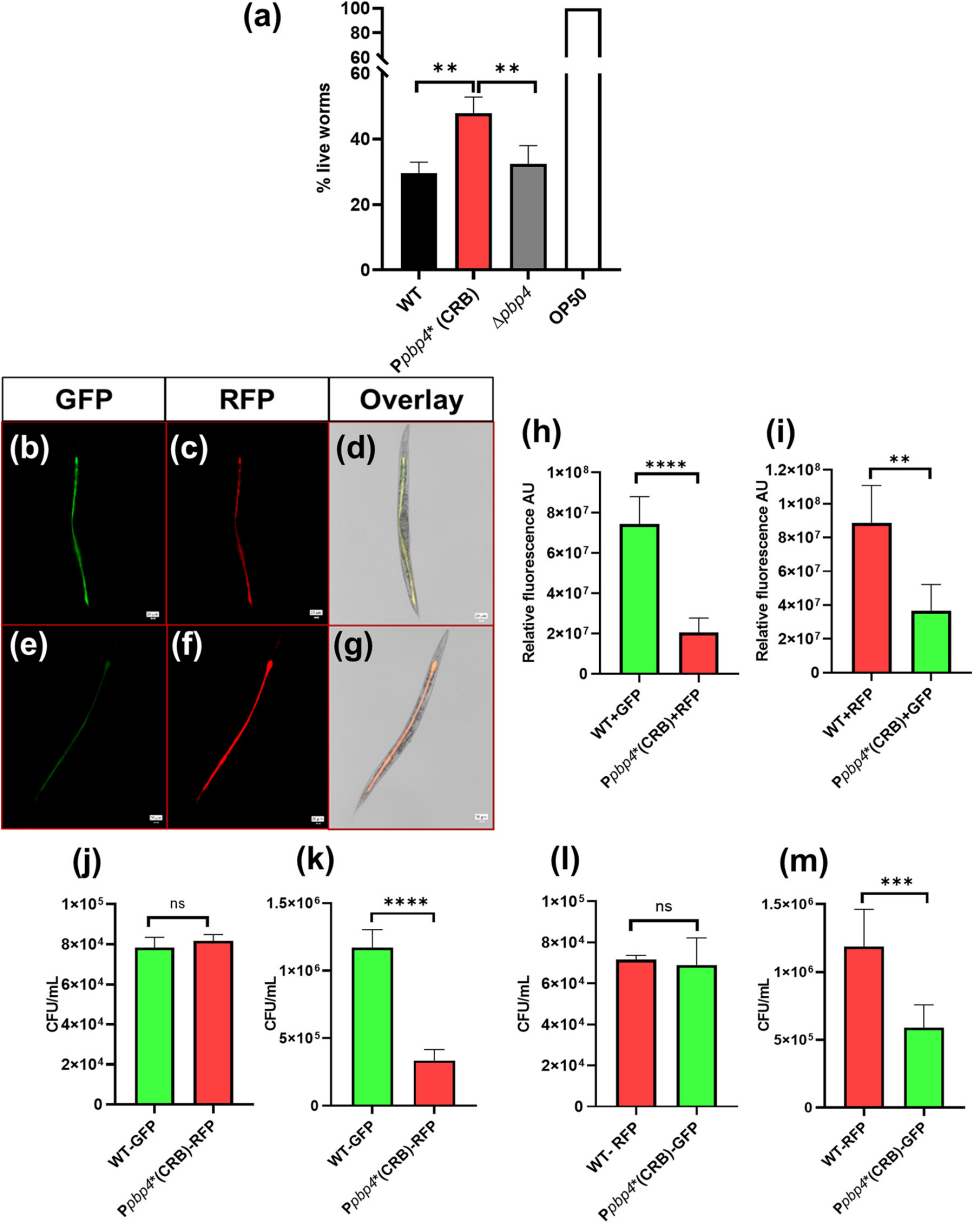
Cells with overexpressed PBP4 have decreased virulence. (a) C. elegans infection assay. Killing assay with isogenic strains demonstrated that SF8300 WT and SF8300 Δ*pbp4* strains had decreased worm survival (30%) compared with SF8300 Ppbp4* (CRB), which had increased worm survival (55%) indicating decreased S. aureus virulence (SF8300 WT versus SF8300 Ppbp4* [CRB], *P* = 0.0021; SF8300 Ppbp4* [CRB] versus SF8300 Δ*pbp4*, *P* = 0.052; SF8300 WT versus SF8300 Δ*pbp4*, *P* = ns). OP50 is an E. coli control strain, which is used as a food source to culture and maintain C. elegans. Fluorescence microscopy. Microscopy for C. elegans infected with GFP- or RFP-expressing isogenic strains demonstrated that SF8300 Ppbp4* (CRB) had a significantly decreased ability to colonize C. elegans compared with WT, after 3 days of infection using a 20× lens objective. Scale bar = 20 μm. (b) Infection of C. elegans with SF8300 WT + GFP. (c) Infection of C. elegans with SF8300 Ppbp4* (CRB) + RFP. (d) Merged image of C. elegans infected with SF8300 WT + GFP and SF8300 Ppbp4* (CRB) + RFP. (e) Infection of C. elegans with SF8300 WT + RFP. (f) Infection of C. elegans with SF8300 Ppbp4* (CRB) + GFP. (g) Merged image of C. elegans infected with SF8300 WT + RFP and SF8300 Ppbp4* (CRB) + GFP. (h) Analysis of RFP and GFP signals following fluorescence microscopy of C. elegans infected with an equal number of SF8300 WT + GFP and SF8300 Ppbp4* (CRB) + RFP demonstrated that there was a significantly increased GFP signal compared to RFP signal from within the worms analyzed, indicating that there was increased colonization of the WT strain compared with Ppbp4* (CRB). For each well, 5 worms were imaged and analyzed. SF8300 WT + GFP versus SF8300 Ppbp4* (CRB) + RFP, *P* < 0.0001. (i) Equal number of SF8300 WT + RFP and SF8300 Ppbp4* (CRB) + GFP demonstrated that there was significantly increased RFP signal compared with GFP signal from within the worms analyzed, indicting that there was increased colonization of the WT strain compared with Ppbp4* (CRB). For each well, 5 worms were imaged and analyzed. SF8300 WT + RFP versus SF8300 Ppbp4* (CRB) + GFP, *P* = 0.0026. CFU determination. (j) Inoculum on day 0, before C. elegans infection with SF8300 WT + GFP and SF8300 Ppbp4* (CRB) + RFP demonstrated that cells of each strain were used in similar proportions. SF8300 WT + GFP versus SF8300 Ppbp4* (CRB) + RFP, *P* = ns. Ten worms were lysed for each condition, and the experiment was performed in triplicates. (k) Plating of bacteria obtained from the gut of C. elegans after 3 days of infection with bacteria indicated that there was a higher proportion of GFP-expressing SF8300 WT CFU than RFP-expressing SF8300 Ppbp4* (CRB) CFU. SF8300 WT + GFP versus SF8300 Ppbp4* (CRB) + RFP, *P* < 0.0001. Ten worms were lysed for each condition, and the experiment was performed in triplicates. (l) Inoculum on day 0, before C. elegans infection with SF8300 WT + RFP and SF8300 Ppbp4* (CRB) + GFP demonstrated that cells of each strain were used in similar proportions. SF8300 WT + RFP versus SF8300 Ppbp4* (CRB) + GFP, *P* = ns. Ten worms were lysed for each condition, and the experiment was performed in triplicates. (m) Plating of bacteria obtained from the gut of C. elegans after 3 days of infection with bacteria indicated that there was a higher proportion of RFP-expressing SF8300 WT CFU than GFP-expressing SF8300 Ppbp4* (CRB) CFU. SF8300 WT + RFP versus SF8300 Ppbp4* (CRB) + GFP, *P* = 0.0001. Ten worms were lysed for each condition, and the experiment was performed in triplicates.

Along with a role in β-lactam resistance, PBP4 has also been associated with pathogenesis. However, its exact role, if any, remains undetermined, as there have been contrasting reports regarding the role of PBP4 in pathogenesis (15, 16). We attempted to understand the role of PBP4 in pathogenesis under wild-type and overexpressed conditions using a C. elegans infection model. We selected one representative strain containing promoter-associated mutations, namely, SF8300 Ppbp4* (CRB) and used it to perform infection studies with C. elegans. Age-synchronized worms were infected with 1.5 × 10^5^ bacteria and were incubated for 3 days, following which they were assessed for worm survival. Worms that responded to mechanical stimulus were considered live, whereas worms that did not respond were counted as dead. Compared with worms infected with WT cells, where the survival rates for the worms were approximately 30%, worms infected with Ppbp4* (CRB) had significantly higher survival rates (55%) (SF8300 WT versus SF8300 Ppbp4* [CRB], *P* = 0.0021) (Fig. 3a), indicating that the presence of promoter-associated mutations led to the decreased killing and thus decreased virulence in C. elegans. Infection with SF8300 Δ*pbp4* also resulted in decreased worm survival, similar to the results obtained by infection with WT cells (SF8300 Ppbp4* [CRB] versus SF8300 Δ*pbp4*, *P* = 0.052). The Escherichia coli strain OP50 was used as a control, where worms displayed 100% survival, indicating that the killing detected was due to S. aureus virulence. Killing assays performed with SF8300ex background strains had similar survival patterns. The inclusion of *abcA* mutants did not affect the C. elegans killing pattern, as both strains with promoter mutations, i.e., SF8300ex Ppbp4* (CRB) and SF8300ex Ppbp4* (CRB) *abcA*::Tn, had increased worm survival compared with strains with wild-type promoters (SF8300ex and SF8300ex Ppbp4* [CRB], *P* = 0.0045; SF8300ex *abcA*::Tn and SF8300ex Ppbp4* [CRB] *abcA*::Tn, *P* = 0.0081) (see Fig. S5 in the supplemental material).

We carried out further experiments with C. elegans infection to determine why the Ppbp4* (CRB) strain showed increased survival. SF8300 WT and SF8300 Ppbp4* (CRB) strains were introduced with constitutively expressing GFP and RFP, respectively, via the constitutively expressing plasmid *pTX_Δ_*, thus generating strains SF8300 WT + GFP and SF8300 Ppbp4* (CRB) + RFP. A competition-killing assay was performed, where an equal number of SF8300 WT and SF8300 Ppbp4* (CRB) cells were used to infect worms. After 3 days of infection, fluorescence microscopy was carried out where the GFP and RFP signals from within the gut of each worm were measured. On a subsequent analysis of the fluorescent signals, it was observed that there was a significantly increased GFP signal detected compared with RFP (Fig. 3b to d and h). This finding indicated that there was a higher proportion of SF8300 WT cells colonized within the gut of C. elegans than SF8300 Ppbp4* (CRB) cells (SF8300 WT + GFP versus SF8300 Ppbp4* [CRB] + RFP, *P* < 0.0001). The experiment was repeated by interchanging the plasmids containing fluorescent proteins, i.e., with the strains SF8300 WT + RFP and SF8300 Ppbp4* (CRB) + GFP (Fig. 3e to g and i). Here, an increased RFP signal compared with GFP signal within the gut of C. elegans was detected, ensuring that it was due to the increased colonization of SF8300 WT and not due to a result of a potential anomaly of using fluorescent proteins (SF8300 WT + RFP versus SF8300 Ppbp4* [CRB] + GFP, *P* = 0.0026).

Before C. elegans were infected with bacteria containing fluorescent proteins, the initial inoculum was plated onto tetracycline-containing TSA plates (Fig. 3j). After it was determined that the initial inoculum contained a similar number of each of the bacterial strains (SF8300 WT + GFP versus SF8300 Ppbp4* [CRB] + RFP, *P* = ns), C. elegans were subjected to lysis after 3 days of infection following which the bacteria accumulated within the gut of the worms were enumerated by performing serial dilutions of the lysate and plating them. There was a significantly higher number of GFP-expressing colonies (representing SF8300 WT) on the plate than that of RFP-expressing colonies (representing SF8300 Ppbp4* [CRB]) (Fig. 3k) (SF8300 WT + GFP versus SF8300 Ppbp4* (CRB) + RFP, *P* < 0.0001). When plasmids were interchanged, there were increased RFP-expressing colonies (representing SF8300 WT) and decreased GFP-expressing colonies (representing SF8300 Ppbp4* [CRB]) (Fig. 3l and m). Together, the C. elegans experiments indicated that the SF8300 Ppbp4* (CRB) was unable to colonize the C. elegans gut as well as SF8300 WT, leading to decreased virulence.

## DISCUSSION

MRSA is one of the most prominent agents contributing to the significant global antimicrobial resistance burden today (3). However, in recent years, the presence of β-lactam resistance in S. aureus strains without *mecA* has been reported (10, 24, 25). In previous studies, we have demonstrated the ability of PBP4 to mediate high-level β-lactam resistance via protein overexpression due to promoter-associated mutations in non-*mecA* strains (7, 17). In this study, we saw that PBP4 could mediate β-lactam resistance independent of PBP2a. As seen by the growth assays, both PBP2a and PBP4 contributed toward β-lactam resistance, as cells containing the promoter-associated mutations were able to survive the β-lactam challenge more significantly than strains that contained only PBP2a, i.e., the WT strains. This result indicated that, when present, PBP4 could supplement the action of PBP2a, causing a further increase in resistance and potentially leads to complications in treatment. Current clinical diagnostic and therapeutic protocols are based on the categorization of the infecting strains as MRSA or MSSA, as the treatment for MRSA is more aggressive than that of MSSA (26). However, due to the rise in *pbp4*-associated resistance, it is likely that targeting only PBP2a for diagnosis and treatment protocols may not suffice.

PBP4 is a low-molecular-weight (LMW) PBP in S. aureus (27). LMW PBPs in other bacteria, such as E. coli (28), Bacillus subtilis (29), Listeria monocytogenes (30), and Streptococcus pneumoniae (31), have been described to possess carboxypeptidase activity, allowing them to maintain the degree of cell wall cross-linking. PBP4 in S. aureus has transpeptidase activity along with carboxypeptidase activity, giving it the ability to perform increased, secondary cell wall cross-linking compared with other bacteria (32). Increased PBP4 expression due to promoter-associated mutations leads to a further increase in cross-linking (17, 33), indicating PBP4’s propensity toward transpeptidase activity over carboxypeptidase activity. This information demonstrates the potential of PBP4 from S. aureus to be a powerful player in β-lactam resistance. The importance of PBP4 in β-lactam resistance was further reiterated when we saw that the promoter-associated mutations studied here led only to *pbp4* upregulation and not *abcA*. AbcA can export various antimicrobial agents, making it an important mediator of antibiotic resistance (14, 19). However, based on the qRT-PCR analysis and hemolysis assay, it was clear that AbcA did not play any role in the resistant phenotype associated with the promoter mutations and that it was all attributed to PBP4 overexpression. It is likely that the presence of mutations possibly led to altered binding of transcriptional factors to the regulatory region, leading to overexpression of PBP4. Reports have suggested that *pbp4* and *abcA* regulation are independent of each other (14); however, since the studied mutations also caused the downregulation of *abcA*, it appears that the regulation of both of these genes are at least partially interdependent, and they potentially share some of their regulatory sequences as well as some regulatory factors. Along with AbcA, S. aureus also has another known exporter of PSMs, namely, the Pmt system (21). It is likely that Pmt compensated for the export of PSMs during *abcA* downregulation, allowing for similar levels of hemolytic activities between various strains. *abcA* mutants did not affect the growth phenotypes in the presence of β-lactams, which indicated that downregulation of *abcA* also did not contribute to resistance (Fig. S3).

In S. aureus, PBP4 maintains the level of peptidoglycan cross-linking by its transpeptidase and carboxypeptidase properties, thus striking a balance between appropriate cross-linking of peptidoglycan monomers and allowing for anchoring of surface proteins that are necessary for S. aureus virulence (34). Our previous studies demonstrated that this well-balanced cell wall dynamic was disrupted in the form of increased cell wall cross-linking due to the overexpression of PBP4 (7, 33). However, the effect of this disruption on the abundance of surface-anchored proteins is unknown. As these surface proteins play vital roles in host attachment, colonization, and infection, we examined the virulence of a strain containing promoter-associated mutations using C. elegans, as this strain exhibited increased PBP4 expression. C. elegans has proven to be a useful infection model system to use to study host-pathogen interactions and has given multiple clues about host response to pathogenic bacteria (35). S. aureus cells mediate C. elegans killing by accumulating within the gut of the worm, leading to intestinal distension and subsequent worm death (36). Based on our results, strains with overexpressed PBP4 were unable to colonize within the gut of the worms to a sufficient enough degree to cause high levels of accumulation and worm death compared with the WT cells. The *abcA* mutants did not change the survival pattern of C. elegans on infection, suggesting that AbcA likely did not have a role in the virulence of C. elegans (Fig. S4). Gut epithelial cells of C. elegans have been described to have similarities with human intestinal epithelial cells, suggesting the possibility that strains containing increased PBP4 expression may have a decreased ability to colonize humans, as S. aureus has been shown to also colonize and infect human intestinal cells, making this study biologically relevant (37). It is likely that the inability of Ppbp4* (CRB) cells to colonize was due to a decrease in surface-anchored proteins due to increased PBP4-mediated cross-linking of the peptidoglycan. In S. aureus, repeating monomers of the peptidoglycan consisting of penta-peptide stems are cross-linked via a penta-glycine bridge (38). The terminal glycine of this pentaglycine bridge forms a peptide bond with the d-alanine of the penta-peptide stem, via the transpeptidase activity of PBP4 (39). However, this glycine is also the site at which sortase A-mediated anchoring of cell surface proteins takes place (40). Due to the PBP4-mediated increase of cell wall cross-linking, we hypothesize that there are not sufficient free glycine sites available for the sortase A-mediated attachment of surface proteins, thus leading to decreased virulence (see Fig. S6 in the supplemental material). Further studies are required to understand the biology that leads to the decreased virulence phenotype of S. aureus strains having *pbp4*-promoter associated mutations, which can potentially help in exploiting this phenomenon for treatment purposes.

## MATERIALS AND METHODS

### Bacterial strains and plasmids.

S. aureus strains were all cultured at 37°C in tryptic soy broth (TSB), with agitation at 180 rpm. Promoter-associated mutations in S. aureus were introduced via splice-overlap PCR and allelic replacement as described previously (41), using the plasmid pJB38 (42). The GFP and RFP encoding regions were amplified from the integration plasmids pGFP-F and pRFP-F, respectively (42), and were cloned into the constitutively expressing plasmid *pTX_Δ_* as described previously (17). The plasmid was introduced into RN4420 by electroporation, following which they were introduced in SF8300 or SF8300 Ppbp4* (CRB) via phage transduction using ϕ11. All strains, primers, and plasmids used in this study are listed in Table 1, 2, and 3, respectively.

**TABLE 1:** Strains used in this study

Strain	Notes	Reference
E. coli DH5α		Invitrogen
RN4220	Laboratory S. aureus strain	BEI Resources
SF8300	USA300 MRSA clinical isolate	9
SF8300 Δ*pbp4*	SF8300 devoid of *pbp4*	9
SF8300 Ppbp4* (CRB)	SF8300 with CRB promoter mutations	This study
SF8300 Ppbp4* (CRT)	SF8300 with CRT promoter mutations	
SF8300 Ppbp4* (Strain 1)	SF8300 with Strain 1 promoter mutations	This study
SF8300 + p*Tx_Δ_* (GFP)	SF8300 with constitutively expressing GFP	This study
SF8300 Ppbp4* (CRB) + p*Tx_Δ_* (GFP)	SF8300 Ppbp4* (CRB) with constitutively expressing GFP	This study
SF8300 + p*Tx_Δ_* (RFP)	SF8300 with constitutively expressing GFP	This study
SF8300 Ppbp4* (CRB) + p*Tx_Δ_* (RFP)	SF8300 Ppbp4* (CRB) with constitutively expressing RFP	This study
SF8300ex	SF8300 devoid of *mecA* gene and *blaZ* plasmid	9
SF8300ex Ppbp4* (CRB)	SF8300ex with CRB promoter mutations	9
SF8300ex *abcA*::Tn	SF8300ex with the *abcA* transposon insertion	This study
SF8300ex Ppbp4* (CRB) *abcA*::Tn	SF8300ex Ppbp4* (CRB) with the *abcA* transposon insertion	This study
SF8300ex Δ*pbp4*	SF8300ex devoid of *pbp4*	9

**TABLE 2:** Primers used in this study

Primer	Sequence (5′–3′)	Use
PBP4-P1	GGGGACAAGTTTGTACAAAAAAGCAGGCTAGTTTGCAATTTCAGATTGTGTACTTGTCGATATCTTTTGCATAATACGACC	*pbp4* deletion using pJB38
PBP4-P2	AAAGCGTTAATCTTCCCTTTTCCAATTCTTAAATATTCCCTAAAAGC	*pbp4* deletion using pJB38
PBP4-P3	AAAAGGGAAGATTAACGCTTTAACATACTAAAAACGGACAAGTTGCACATTATAAAGCTGCGAAACTTGTCCG	*pbp4* deletion using pJB38
PBP4-P4	GGGGACCACTTTGTACAAGAAAGCTGGGTGAAGATTTTAATAGATATATCACAGAAATTATGAAAATAAGACAACG	*pbp4* deletion using pJB38
Ppbp4*-pImay-NotI-for	AGTTTGCGGCCGCAGATTGTGTACTTGTCGATATCTTTTG	Cloning of P*pbp4* into the plasmid pImay
Ppbp4* pImay-kpn1-rev	TCTTGGTACCTTGTTGGTGCAAATGTACGTAATCTTG	Cloning of P*pbp4* into the plasmid pImay
Ppbp4* (CRB)-rev	ACAAAAAATGCAATAGAAATATTCTATCATATAAATGTTATGAGCGGTATTTTG	Introduction of CRB mutations in Ppbp4*
Ppbp4* (CRB)-for	ATATTTCTATTGCATTTTTTGTATTTATATGATAGAATATTTCTATTGC	Introduction of CRB mutations in Ppbp4*
Ppbp4*-(CRT)-kpn1-rev	ATTGGTACCAGATACTGTAATTTGTAATAGGTCTGCGATTG	Introduction of CRT mutations in Ppbp4*
Ppbp4*_Strain 1_for	TATATGATAGAATATTTCTATAGCATTTTTTG	Introduction of Strain 1 mutations in Ppbp4*
Ppbp4*_Strain 1_rev	ATTACAAAAAATGCtATAGAAATATTCTATC	Introduction of Strain 1 mutations in Ppbp4*
GFP_BamHI_FP	AGGATCCTAAAAAGTGAATAGAGGTGGAATAATGTCAAAAGGAGAAGAATTATTTACAG	Cloning GFP into p*Tx_Δ_*
GFP_MluI_RP	ATACGCGTTTACTTATATAATTCATCC	Cloning GFP into p*Tx_Δ_*
RFP_BamH1_FP	ATATGGATCCTGATTAACTTTATAAGGAGG	Cloning RFP into p*Tx_Δ_*
RFP_MulI_RP	ATACGCGTTTATAAAAACAAATGATGACG	Cloning RFP into p*Tx_Δ_*
NMC-Tn-upstream	CTCGATTCTATTAACAAGGG	Transposon insertion verification
abcA-1	TTAATCTGTTAATTTTTGAGACACTAC	Transposon insertion verification
pbp4-for	TTGATTTAATGAATAACAAAGCTAAAGC	qRT-PCR for *pbp4*
pbp4-rev	AGTCTCTAGCAGTCGTTACAGTACGTTC	qRT-PCR for *pbp4*
abcA-for	ACCAATTTCAGGTATAGTTATGTTGCTAAC	qRT-PCR for *abcA*
abcA-rev	TGGAAAGATTGATTAAAGGCATAGATAAC	qRT-PCR for *abcA*
gyrB-for	ATTGCTCTAGTAAAAGTCCTGAAGAATG	qRT-PCR for *gyrB*
gyrB-rev	TAATCGTGCTTTTTCAACATTTAATATC	qRT-PCR for *gyrB*

**TABLE 3:** Plasmids used in this study

Plasmid	Notes	Reference
pImay + P*pbp4* (CRB)	Insertion of P*pbp4* promoter mutation	This study
pJB38 + P*pbp4* (CRT)	Insertion of P*pbp4* promoter mutation	This study
pJB38 + P*pbp4* (Strain 1)	Insertion of P*pbp4* promoter mutation	This study
pJB38 + Δ*pbp4*	Deletion of *pbp4*	
p*Tx_Δ_* + GFP	Constitutive expression of GFP	This study
p*Tx_Δ_* + RFP	Constitutive expression of RFP	This study

### Growth curve assays.

Growth assays were performed using the automated microbiology growth curve analysis system Bioscreen C (Growth Curves USA). Overnight cultures of bacteria were diluted to an optical density (OD) of 0.1 in TSB with or without antibiotics, and 200 μL was added to each well of a honeycomb Bioscreen C plate in triplicates. The assay was carried out for 20 h with continuous orbital shaking at 37°C. Each condition was tested in triplicate, and the experiment was performed twice to ensure reproducibility.

### Immunoblotting.

Overnight cultures of bacteria were subcultured into 50-mL flasks containing TSB such that the initial OD at 600 nm (OD_600_) of the flasks was 0.1. The cells were cultured to an OD_600_ of 1, following which cells were collected and resuspended in phosphate-buffered saline (PBS) containing CompleteMini protease inhibitor cocktail (Roche). The cells were mechanically lysed using the FastPrep (MP Biochemicals) instrument, and whole-cell lysates were obtained. The cell membrane fraction was isolated from the lysates by performing ultracentrifugation at 66,000 × *g* for 1 h (Sorvall WX Ultra 80 centrifuge; Thermo Fisher Scientific). After the obtained pellet was resuspended with PBS, protein estimation was carried out using the Pierce BCA protein assay kit (Thermo Fisher). The samples were separated by performing SDS-PAGE on a 10% gel, following which they were transferred onto a low-fluorescence polyvinylidene difluoride (PVDF) membrane (Millipore). Blocking was performed for 1 h (5% skimmed milk in Tris-buffered saline containing 0.5% Tween), primary antibody staining was carried out overnight at 4°C (polyclonal anti-PBP4, custom antibody from Thermo Fisher, 1:1,000), and secondary antibody staining was performed using an anti-rabbit antibody (Azure anti-rabbit NIR700, 1:20,000 dilution). The blots were imaged using the Azure C600 imager, and analysis was performed using ImageJ.

### qRT-PCR.

Overnight cultures of bacteria were subcultured into 50-mL flasks containing TSB such that the initial OD_600_ of the flasks was 0.1, and cells were allowed to grow for 4 h, at which point 5 × 10^9^ bacterial cells were harvested and washed. Cells were lysed using the FastPrep (MP Biochemicals) instrument following which RNA isolation was performed using the Qiagen RNeasy minikit. On confirmation of RNA quality, cDNA synthesis was performed using the SuperScript IV reverse transcriptase (RT) kit. qRT-PCR was performed using SYBR PCR Mastermix in an ABI 7500 system (Applied Biosystems) and primers for *pbp4*, *abcA*, and the housekeeping gene *gyrB*.

### Butanol extraction of PSMs.

PSMs were extracted from culture supernatants as described previously (21, 43). Briefly, overnight cultures of bacteria were subcultured into 50-mL flasks containing TSB such that the initial OD_600_ of the flasks was 0.1. After 24 h, the cells were collected and centrifuged for 30 min, and 30 mL of supernatant from each strain was collected to which 10 mL 1-butanol was added. The samples were mixed by shaking at 180 rpm for 2 h at 37°C. Samples were then centrifuged, and 7 mL of the upper layer from each sample was collected. The samples were dried using a vacuum centrifuge (Eppendorf), and the dried pellet was resuspended in 8 M urea. Samples were diluted 10-fold for hemolysis experiments.

### Hemolysis assay.

Two percent sheep blood was prepared using chilled PBS and was washed twice to get rid of lysed erythrocytes. After a washing step, 100 μL of the blood was added to a 96-well round-bottom plate, to which 100 μL of samples of PSMs extracted with butanol was added in decreasing concentrations (from 1/10th to 1/1,280th of the sample). The plates were incubated for 1 h at 37°C, following which they were centrifuged at 1,500 rpm for 10 min. A total of 100 μL of the supernatant was collected and transferred onto a flat-bottom 96-well plate. Absorbance was measured at 540 nm using a SpectraMax M5 (Molecular Devices) instrument. A total of 0.5% Triton was used as a control.

A total of 2 μL of butanol-extracted PSMs was spotted onto TSA-blood plates (5% sheep blood) for each sample. Urea was spotted as a negative control and 0.5% Triton was spotted as a positive control. After samples were dried, plates were incubated at 37°C overnight.

### C. elegans-S. aureus infection assays.

C. elegans worms were obtained from the Caenorhabditis Genetics Center (CGC). The liquid killing assay was performed as described previously (44), with certain modifications. The C. elegans strain used in this study was DH26 [rrf-3(b26) II], a temperature-sensitive, spermatogenesis-defective strain. Briefly, adult hermaphrodites were age synchronized (45) using household bleach and 5 N NaOH and allowed to grow on nematode growth medium (NGM) plates for 40 h at 26°C. Fifteen young adult worms were picked from this plate and added to 96-well plates containing 100 μL liquid killing media (80% M9 buffer, 20% TSB, 100 μg/mL cholesterol, and 7.5 μg/mL nalidixic acid) and 1.5× 10^5^ S. aureus cells. Wells containing OP50 in media (80% M9 buffer, 20% LB, and 200 μg/mL cholesterol) were included as controls. The plate was incubated at 26°C for 3 days following which live and dead worms were counted. The experiment was performed thrice, in triplicates in order to ensure reproducibility.

For the competition assay, WT + GFP and Ppbp4* (CRB) + RFP cells were mixed in equal amounts before 1.5× 10^5^ cells were added to each well. The media also contained 12.5 μg/mL tetracycline, which is the selection antibiotic for the *pTX_Δ_* plasmid.

### Fluorescence microscopy and image analysis.

Fluorescence microscopy and analysis were performed as described previously (46). After 3 days of infection with S. aureus, worms were transferred from the wells to 1.5-mL microcentrifuge tubes. The worms were washed with M9 buffer containing 10 mM sodium azide, following which they were treated with 100 μg/mL gentamicin for 30 min, twice. Following the gentamicin treatment, worms were washed thrice with M9 buffer containing 10 mM sodium azide. The worms were then placed onto an agar pad (2% agar) on glass slides, and fluorescence microscopy was performed using the Keyence BZ-X800 all-in-one fluorescence microscope using the 20× objective. The exposure time and all other settings remained the same for all samples.

The BZX analyzer software was used to measure fluorescence intensities within a worm. After stitching images wherever necessary, the “hybrid cell count” function was used to select the area of the worm and measure fluorescence intensities for GFP and RFP. For each condition, at least 5 worms were imaged and analyzed.

### Gut CFU determination.

Gut bacterial enumeration was performed as described previously (47). After the gentamicin treatment and wash steps as described above, the worms were resuspended in 250 μL M9 buffer containing 10 mM sodium azide, mixed with 200 mg of 1-mm zirconia beads (Research Products International), and lysed by vortexing the tubes for 2 min. Before lysis, 20 μL of the supernatant was collected for plating. Following lysis, samples were diluted to 10^−4^, plated onto TSA plates containing tetracycline, and incubated at 37°C. The next day, colonies were counted based on the color they expressed. The experiment was performed twice, in triplicates to ensure reproducibility. Each tube contained 10 worms, and the experiment was performed twice, in triplicates.

### Sequencing.

The fidelity of all the mutants and plasmid constructs was validated through Sanger sequencing (Eurofins Genomics, USA).

### Bioinformatics and statistical analysis.

Statistical analyses were performed using GraphPad Prism. Comparisons between groups were analyzed by two-tailed Student’s *t* test whenever stated. DNA sequence analysis was performed using DNAstar software.
