# Supplementary material for: A Nonclassical Mechanism of β-Lactam Resistance in Methicillin-Resistant Staphylococcus aureus and Its Effect on Virulence
Source: Microbiol Spectr. 2022 Oct 31;10(6):e02284-22. doi: 10.1128/spectrum.02284-22 (PMC9769611; doi:10.1128/spectrum.02284-22)
Supplement: Supplemental file 1 — Supplemental methods and Fig. S1 to S6. Download spectrum.02284-22-s0001.pdf, PDF file, 0.6 MB [file spectrum.02284-22-s0001.pdf]

A non-classical mechanism of β-lactam resistance in Methicillin-Resistant *Staphylococcus aureus* (MRSA) and its effect on virulence

<u>Supplementary file</u>

<u>Methods</u>

Generation of *abcA* mutants

Bocillin assay

<u>Figures</u>

Figure S1: Loading control for figure 1(b).

Figure S2: Effect of promoter mutations on downstream genes.

Figure S3: Phenotypic verification of SF8300ex background strains.

Figure S4: Effect of *abcA* mutant on resistance.

Figure S5: Effect of *abcA* mutant on virulence.

Figure S6: Increased cell-wall cross-linking due to PBP4 overexpression can result in decreased anchoring of sortase A mediated cell surface associated proteins.

Methods:

Generation of *abcA* mutants

Using phage Φ11, the transposon insert was introduced to SF8300ex and SF8300ex P*pbp4** (CRB) using JE2 *abcA*::Tn (NE1509, Nebraska Transposon mutant SAUSA300_0630) as the donor strain. Presence and location of the transposon was verified by PCR and Sanger sequencing.

Bocillin assay

Overnight cultures of the selected *S. aureus* strains were diluted in TSB such that the OD_{600} value was 0.1. Cells were grown at 37°C, 180 rpm until OD_{600} of 1 after which 50 mL of the culture was collected for centrifugation at 2451 g (3500 rpm) to obtain cell pellets. The pellets were dried and resuspended in 500 µL PBS. For whole cell labeling, cells were incubated with 10 µM Bocillin-Fl (Thermo Fisher) for 30 minutes at 35°C. Cell lysis was carried out using FastPrep-24 mechanical disrupter from MP Biomedical. After centrifugation, the supernatant was collected and protein estimation was carried out using Pierce BCA Protein Assay kit (Thermo Fisher). Protein samples were boiled for 10 minutes in sample buffer and 20 µg of protein was loaded onto a 10% SDS-polyacrylamide gel and electrophoresis was carried out at 80V. The bands were visualized using the Typhoon 9410 imager (Amersham/GE Healthcare).

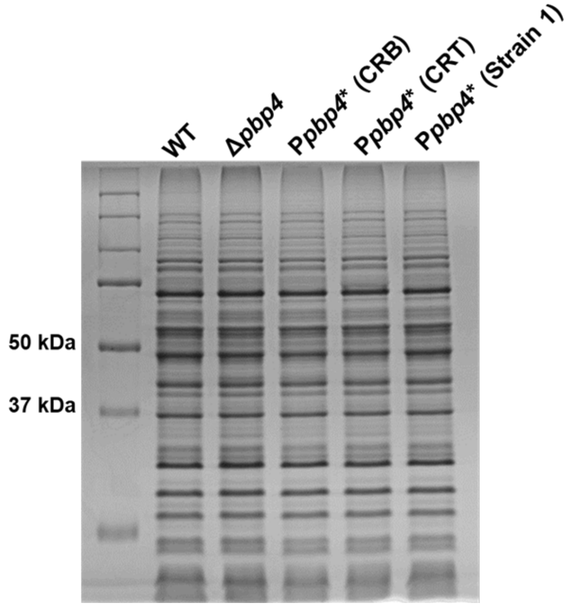

Figure S1: Loading control for figure 1(b). SDS-PAGE followed by staining with Coomassie Blue.

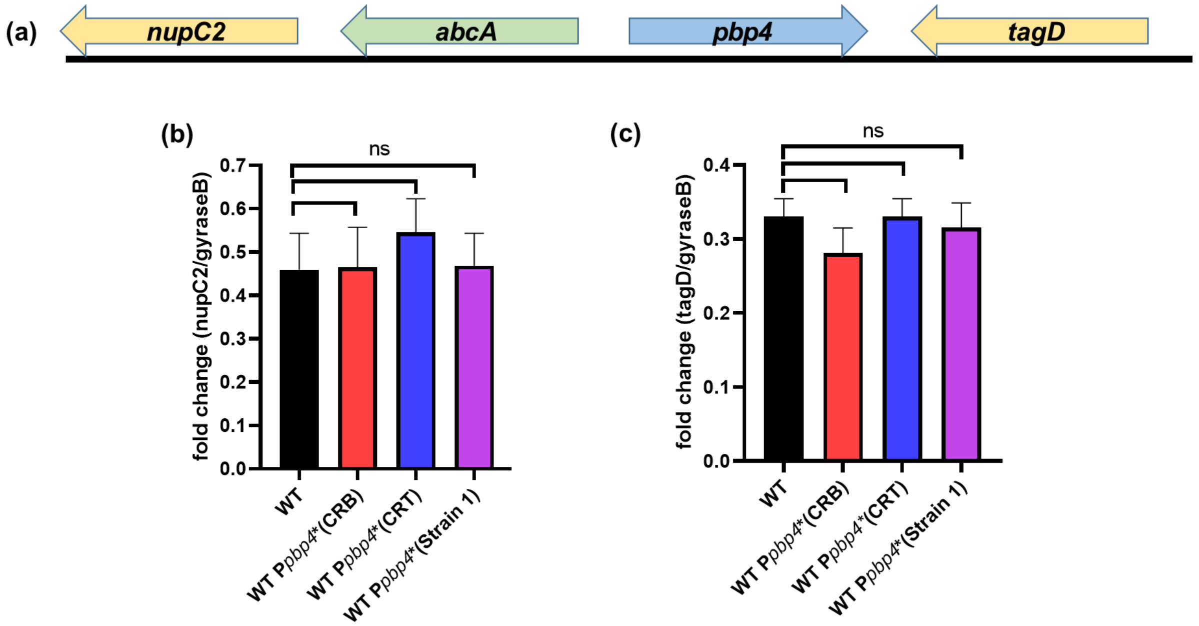

Figure S2: Effect of promoter mutations on downstream genes.

(a) Schematic representation of the genes flanking *abcA* and *pbp4*.

qRTPCR analysis of **(b)** nupC2 and **(c)** tagD indicated that the expression of either of the genes is not affected by the presence of *pbp4*/*abcA* promoter-associated mutations. SF8300 WT versus SF8300 P*pbp4** (CRB), SF8300 WT versus SF8300 P*pbp4** (CRT), SF8300 WT versus SF8300 P*pbp4** (Strain 1), P-value = ns.

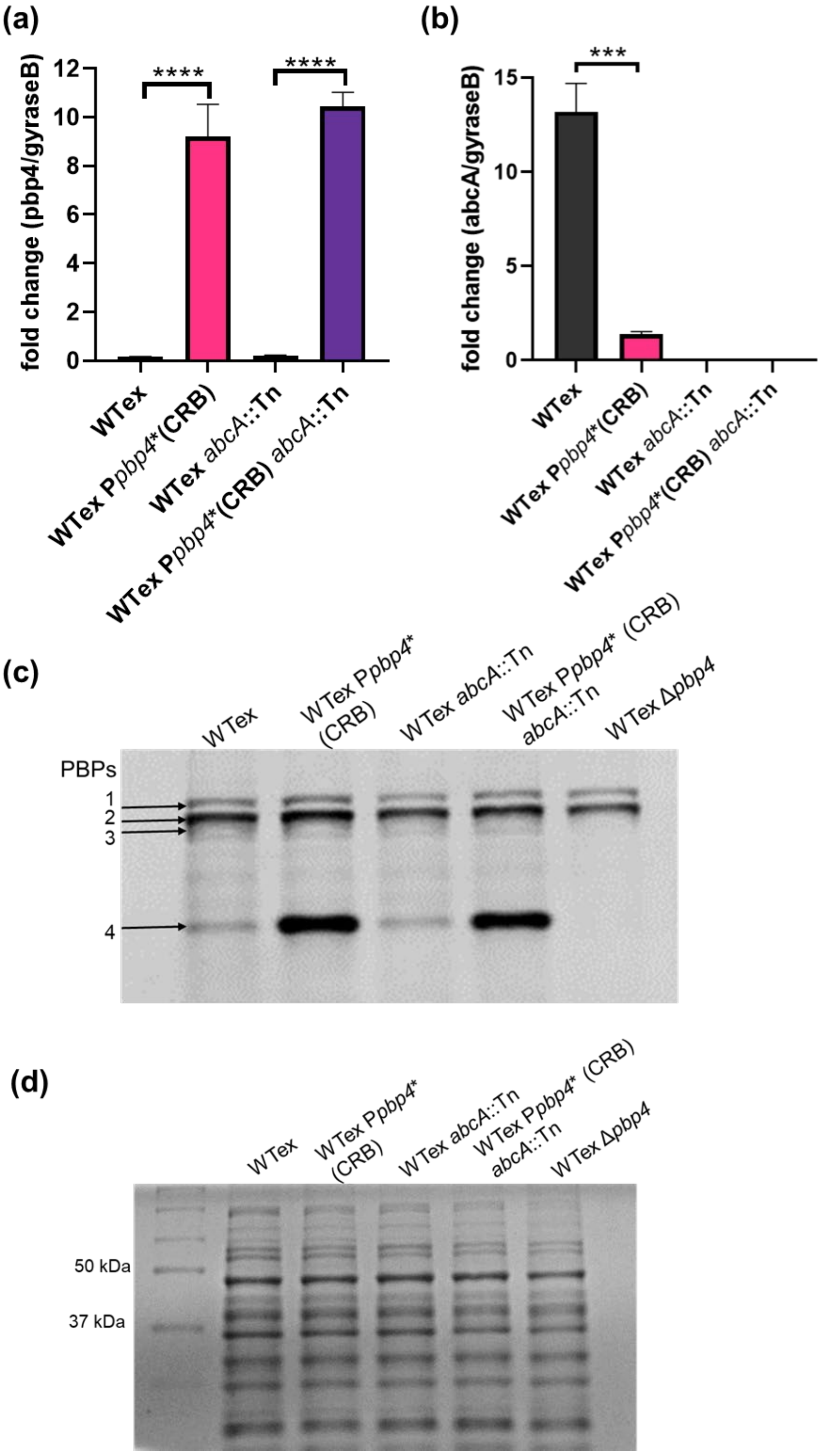

Figure S3: Phenotypic verification of SF8300ex background strains.

(a) qRTPCR analysis of *pbp4* expression in SF8300ex background strains. Transcriptional expression of *pbp4* was significantly higher in strains containing promoter mutations,

namely SF8300ex P*pbp4** (CRB) and SF8300ex P*pbp4** (CRB) *abcA*::tn compared to strains with wild-type promoter. SF8300ex and SF8300ex P*pbp4**(CRB), P-value = 0.0003. SF8300ex *abcA*::Tn and SF8300ex P*pbp4**(CRB) *abcA*::Tn, P-value > 0.0001.

(b) qRTPCR analysis of *abcA* expression in SF8300ex background strains. Transcriptional expression of *abcA* was significantly lowered in strains containing promoter mutations, namely SF8300ex P*pbp4** (CRB) compared to SF8300ex. No expression was detected for strains containing the *abcA* transposon (SF8300ex *abcA*::Tn and SF8300ex P*pbp4**(CRB) *abcA*::Tn). SF8300ex and SF8300ex P*pbp4**(CRB), P-value = 0.0007.

(c) Bocillin-Fl assay of SF8300ex background strains. Proteins of the membrane fraction were labelled with 10uM Bocillin-Fl and separated on a 10% SDS-PAGE gel. Strains containing promoter mutations had increased expression of PBP4 (SF8300ex P*pbp4** (CRB) and SF8300ex P*pbp4** (CRB) *abcA*::Tn) compared to strains with wild-type promoter.

(d) Loading control for figure S4 (c). Coomassie Blue staining of the gel used to perform Bocillin-Fl assay as a loading control.

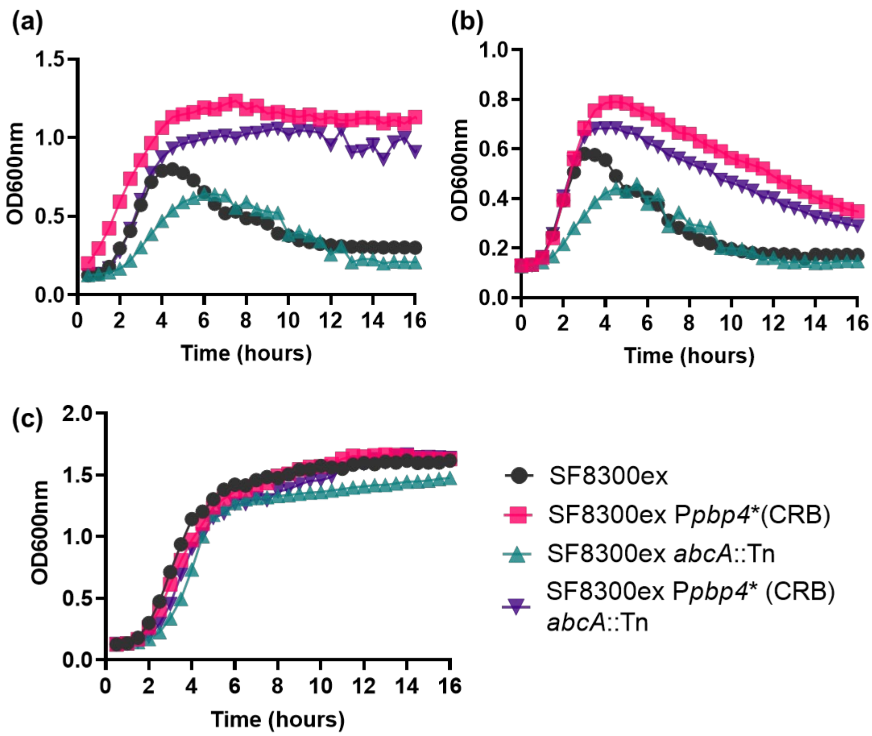

Figure S4: Effect of *abcA* mutant on resistance.

- **(a)** Growth assay demonstrating that strains that were *abcA* mutants namely SF8300ex *abcA*::Tn and SF8300ex P*pbp4**(CRB) *abcA*::Tn had similar growth patterns to strains SF8300ex and SF8300ex P*pbp4**(CRB) respectively, in presence of 0.25 µg/mL nafcillin. SF8300ex *abcA*::Tn and SF8300ex P*pbp4**(CRB) *abcA*::Tn, P-value < 0.0001.
- **(b)** Growth assay demonstrating that strains that were *abcA* mutants namely SF8300ex *abcA*::Tn and SF8300ex P*pbp4**(CRB) *abcA*::Tn had similar growth patterns to strains SF8300ex and SF8300ex P*pbp4**(CRB) respectively, in presence of 0.5 µg/mL oxacillin. SF8300ex *abcA*::Tn and SF8300ex P*pbp4**(CRB) *abcA*::Tn, P-value = 0.0075.
- **(c)** Growth assay in absence of antibiotics.

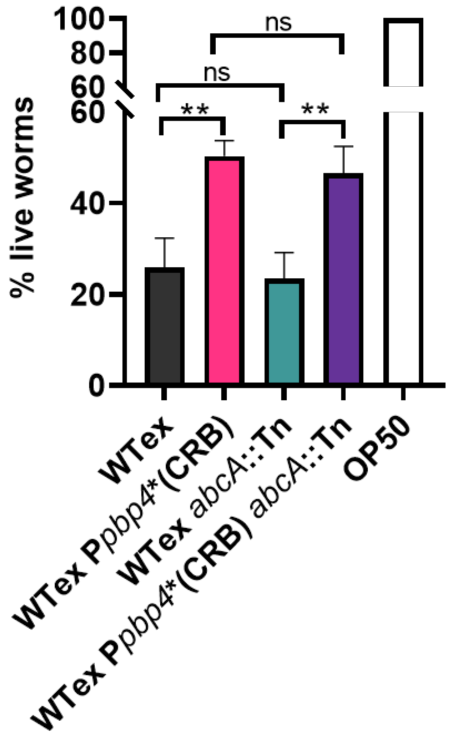

Figure S5: Effect of *abcA* mutant on virulence.

Killing assay of *C. elegans* with SF8300ex (WTex) background strains indicated that *abcA* mutants namely SF8300ex *abcA*::Tn and SF8300ex P*pbp4**(CRB) *abcA*::Tn had similar survival rates as SF8300ex and SF8300ex P*pbp4**(CRB) respectively. SF8300ex and SF8300ex P*pbp4**(CRB), P-value = 0.0045. SF8300ex *abcA*::Tn and SF8300ex P*pbp4**(CRB) *abcA*::Tn, P-value = 0.0081. OP50 is the *E. coli* control.

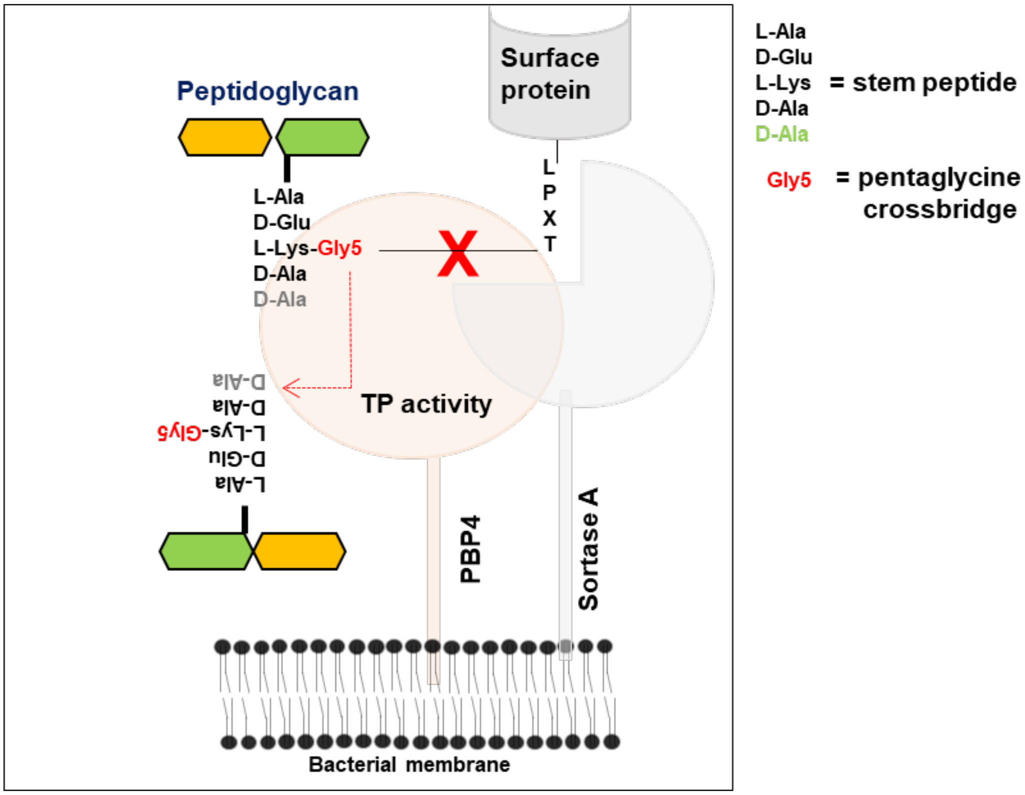

Figure S6: Increased cell-wall cross-linking due to PBP4 overexpression can result in decreased anchoring of sortase A mediated cell surface associated proteins.
